# A deep learning-based ensemble method for helmet-wearing detection

**DOI:** 10.7717/peerj-cs.311

**Published:** 2020-12-07

**Authors:** Zheming Fan, Chengbin Peng, Licun Dai, Feng Cao, Jianyu Qi, Wenyi Hua

**Affiliations:** 1 College of Information Science and Engineering, Ningbo University, Ningbo, China; 2 Ningbo Institute of Industrial Technology, Chinese Academy of Sciences, Ningbo, China

**Keywords:** Ensemble method, Deep learning, Helmet-wearing detection, Face detection

## Abstract

Recently, object detection methods have developed rapidly and have been widely used in many areas. In many scenarios, helmet wearing detection is very useful, because people are required to wear helmets to protect their safety when they work in construction sites or cycle in the streets. However, for the problem of helmet wearing detection in complex scenes such as construction sites and workshops, the detection accuracy of current approaches still needs to be improved. In this work, we analyze the mechanism and performance of several detection algorithms and identify two feasible base algorithms that have complementary advantages. We use one base algorithm to detect relatively large heads and helmets. Also, we use the other base algorithm to detect relatively small heads, and we add another convolutional neural network to detect whether there is a helmet above each head. Then, we integrate these two base algorithms with an ensemble method. In this method, we first propose an approach to merge information of heads and helmets from the base algorithms, and then propose a linear function to estimate the confidence score of the identified heads and helmets. Experiments on a benchmark data set show that, our approach increases the precision and recall for base algorithms, and the mean Average Precision of our approach is 0.93, which is better than many other approaches. With GPU acceleration, our approach can achieve real-time processing on contemporary computers, which is useful in practice.

## Introduction

Helmets can play a vital role in protecting people. For example, many severe accidents in production and work sites and roads have been related to violations of wearing helmets. Some personnel may lack safety awareness in a working site and often do not or forget to wear helmets. On the road, craniocerebral injury is the leading cause of serious injury to cyclists in road traffic (World Health Organization, 2006). However, wearing a helmet reduces the risk of head injury of motorcycle riders by 69% (Liu et al., 2008), and wearing a helmet reduces the risk of head injury for cyclists by 63%–88% (Thompson, Rivara & Thompson, 1999).

Monitoring helmet-wearing manually can have many limitations, as people can be fatigue and costly. Reducing manual monitoring while ensuring that relevant personnel wearing helmets all the time in the working area has become an urgent problem.

Image recognition technology can reduce the workforce and material expenditures, and can significantly protect workers in many areas. Developments of computer vision algorithms and hardware (Feng et al., 2019) have paved the road for the application in helmet detection. Deep neural networks have gained much attention in image classification (Krizhevsky, Sutskever & Hinton, 2017), object recognition (Donahue et al., 2013), and image segmentation (Garcia-Garcia et al., 2017).

Previous computer vision algorithms for helmet detection are usually used in relatively simple scenes. For helmet detection, Rubaiyat et al, (2016) used a histogram of oriented gradient and a support vector machine to locate persons, then used a hough transform to detect helmet for the construction worker. Li et al. (2017b) identified helmets by background subtraction. Li et al. (2017a) used ViBe background modeling algorithm and human body classification framework C4 to select people and heads, and then identified whether people wore helmets through color space transformation and color feature recognition. However, such approaches are typically not suitable for complex scenes and dynamic backgrounds, such as construction sites, workshops, and streets.

Choudhury, Aggarwal & Tomar (2020) and Long, Cui & Zheng (2019) use single shot object detector algorithm to detect helmets. Siebert & Lin (2020) used RetinaNet which uses a multi-scale feature pyramid and focal loss to address the general limitation of one-stage detectors in accuracy, it works well in certain situations but its performance is highly scene dependent and influenced by light. Bo et al. (2019) use the You Only Look Once (YOLO) algorithm to accurately detect helmet wear in images with an average of four targets. However, most of these approaches are not suitable for both small and large helmets at the same time.

In this work, we propose a framework to integrate two complementary deep learning algorithms to improve the ability of helmet-wearing detection in complex scenes. Our approach is able to identify regular-size and tiny-size objects at the same time for helmet-wearing detection, and can be used for detection in complex scenes. This framework can outperform traditional approaches on benchmark data.

## Related Work

The starting point of CNN is the neurocognitive machine model (Fukushima & Miyake, 1982). At this time, the convolution structure has appeared. The classic LeNet (LeCun et al., 1998) was proposed in 1998. However, CNN’s edge began to be overshadowed by models such as SVM (support vector machine) later. With the introduction of ReLU (Rectified Linear Units), dropout, and historic opportunities brought by GPU and big data, CNN ushered in a breakthrough in 2012: AlexNet (Krizhevsky, Sutskever & Hinton, 2017). In the following years, CNN showed explosive development, and various CNN models emerged. CNN has gradually gained the favor of scholars due to its advantages of not having to manually design features when extracting image features (Shi, Chen & Yang, 2019).

Many recent object detection approaches are based on RCNN (Region-based Convolutional Neural Networks) algorithms and YOLO algorithms (Redmon et al., 2016). RCNN is an improved algorithm based on CNN. Girshick et al. propose an epoch-making RCNN algorithm (Girshick et al., 2014) in the field of object detection. The central idea is to use a search selective method to extract some borders from the image. Then the size of the area divided by the border is normalized to the convolutional neural network input size, and then the SVM is used to identify the target. The bounding box of the target is obtained through a linear regression model. It brought deep learning and CNN to people’s sight. However, there are disadvantages such as cumbersome training steps, slow test training speed, and large space occupation.

In order to improve the training and testing speed of RCNN, Fast RCNN algorithm (Girshick, 2015) was developed. It uses fewer layers while adding an ROI pooling layer to adjust the convolution area, and using softmax instead of the original SVM for classification. Compared with RCNN, Fast RCNN has improved training and testing speed. However, because the selective search method is also used to extract the borders of the region of interest, the speed of this algorithm is still not ideal for working with large data sets. Later, Faster RCNN (Ren et al., 2015) integrates feature extraction, proposal extraction, bounding box regression, classification, etc. into a network. The overall performance is far superior to CNN, and at the same time, it runs nearly much faster than CNN. Thus, Faster RCNN is commonly used in many applications. The Faster RCNN performs well for relatively large objects, but when detecting small faces or helmets, there will be a large false negative rate.

Tiny Face has made certain optimizations for small face detection. It mainly optimizes face detection from three aspects: the role of scale invariance, image resolution, and contextual reasoning. Scale invariance is a fundamental property of almost all current recognition and object detection systems, but from a practical point of view, the same scale is not applicable to a sensor with a limited resolution: the difference in incentives between a 300px face and a 3px face is undeniable (Hu & Ramanan, 2017). Ramanan et al. conducted an in-depth analysis of the role of scale invariance, image resolution, and contextual reasoning. Compared with mainstream technology at the time, the error rate can be significantly reduced (Hu & Ramanan, 2017).

Boosting algorithm was initially proposed as a polynomial-time algorithm, and the effectiveness has been experimentally and theoretically proved (Schapire, 1990). Afterward, Freund et al. improved the Boosting algorithm to obtain the Adaboost algorithm (Freund & Schapire, 1997). The principle of the algorithm is to filter out the weights from the trained weak classifiers by adjusting the sample weights and weak classifier weights. The weak classifiers with the smallest coefficients are combined into a final robust classifier.

In this work, in order to identify a variety of heads and helmets in complex scenes, we propose a framework to use incorporate multiple complementary deep learning algorithms to improve the joint performance.

## Materials & Methods

### Method

To address the helmet-wearing detection problem, we compare several object detection methods, such as the naive Bayes classifier, SVM, and Artificial Neural Networks classifier. Naive Bayes usually needs independent distributive premises. SVM is difficult to training for various scenes. In the case of a complex scene and huge training data, artificial neural networks are expected to have better accuracy and reliability, so we propose to use artificial neural networks, especially, convolutional neural networks, to solve this issue. To address the disadvantages raised by long-range cameras, we further improve the performance by integrating multiple complementary deep neural network models.

#### Base algorithms

##### Faster RCNN for detecting faces and helmet-wearing.

After images are fed, Faster RCNN firstly extracts image feature maps through a group of basic conv+relu+pooling layer. Next, RPN (Region Proposal Networks) will set a large number of anchors on the scale of the original image, and randomly select 128 positive anchors and 128 negative anchors from all anchors for binary training, and use these anchors and a softmax function to initially extract positive anchors as the candidate area. At this time, the candidate regions are not accurate and require bounding boxes.

For a given image *I*, we use *A* to represent the ground-truth anchors. We use *A*_*F*_ and *c*_*F*_ to represent the identified bounding boxes and helmet-wearing confidence scores, respectively, computed by the Faster-RCNN algorithm. If we use *F* to represent the algorithm, *W*_*F*_ to represent the weight of the network, this approach can be written as follows.

(1) \begin{eqnarray*}{A}_{F},{c}_{F}=F \left( I,{W}_{F} \right) \end{eqnarray*}

If we consider ${A}_{F}=F \left( I,{W}_{F} \right) [0]$ and ${c}_{F}=F \left( I,{W}_{F} \right) \left[ 1 \right] $, we can use

(2) \begin{eqnarray*}Loss \left( F \left( I,{W}_{F} \right) \left[ 0 \right] ,F \left( I,{W}_{F} \right) \left[ 1 \right] ,A \right) \end{eqnarray*}

to represent the loss function (Fukushima & Miyake, 1982) when to minimize differences between the detected anchors and the ground-truth.

Thus, when we train this model, the optimization is as follows.

(3) \begin{eqnarray*}{W}_{F}^{\ast }={\text{argmin}}_{{\mathrm{ W}}_{\mathrm{F}}}Loss(F(I,{W}_{F})[0],F(I,{W}_{F})[1],A)\end{eqnarray*}

##### Tiny Face for detecting faces.

The overall idea of Tiny Face is similar to RPN in Faster RCNN, which is a one-stage detection method. The difference is that some scale specific design and multi-scale feature fusion are added, supplemented by image pyramid so that the final detection effect for small faces is better. The training data were changed by three scales, with one-third of the probability respectively and sent to the network for training. Multiple scales could be selected to improve the accuracy rate in the prediction.

For a given image *I*, we can also use *A*_*T*_ and *c*_*T*_ to represent the identified bounding boxes and confidence scores computed by the Tiny Face algorithm, so if we use *T* to represent the Tiny Face algorithm and *W*_*T*_ to represent the corresponding weight, we can have

(4) \begin{eqnarray*}{A}_{T},{c}_{T}=T(I,{W}_{T})\end{eqnarray*}

However, Tiny Face can only be used to determine whether the detection target contains a human face and cannot directly distinguish whether the target is wearing a helmet. Thus, we propose to use CNN to overcome this disadvantage.

##### CNN for detecting helmet-wearing.

For anchors determined by Tiny Face, we can use a CNN to detect helmets above the face. We enlarge the face area detected by the Tiny Face and feed it into the CNN model for prediction. The confidence scores indicating whether there is a helmet above the face can be computed by the CNN algorithm

(5) \begin{eqnarray*}{c}_{C}=C({A}_{T},I,{W}_{C}),\end{eqnarray*}

where *C* is a function representing the forward propagation of CNN. Here, *C* is a composition of two convolution layers and one fully connected layer.

The loss function is again to minimize the difference between detected helmets and the ground-truth

(6) \begin{eqnarray*}Loss({A}_{T},C({A}_{T},I,{W}_{C}),A).\end{eqnarray*}

#### Ensemble model detecting high and low resolution helmets

For the two lists of face anchors *A*_*F*_ and *A*_*T*_ detected by the base algorithms above, we merge them with the following strategy. We first initialize an empty anchor list *A*_*S*_ and two score vector *c*_*SF*_ and *c*_*SC*_.

For the *i*th anchor in *A*_*F*_ and the corresponding score in *c*_*F*_, namely, *A*_*F*_[i] and *c*_*F*_[i], we first insert them into *A*_*S*_ and *c*_*SF*_ respectively. Then *A*_*F*_[i] is compared with all the anchors in *A*_*T*_. If some anchors in *A*_*T*_ have more than 60% overlapping area with *A*_*F*_[i], we remove these anchors in *A*_*T*_ and remove the corresponding entries in *c*_*C*_. We also take the mean value of the removed entries in *c*_*C*_ and insert it into *c*_*SC*_. If no overlapped anchors in *A*_*T*_ is found, we insert zero into *c*_*SC*_.

After all the anchors in *A*_*F*_ in processed, the remaining anchors in *A*_*T*_, the remaining confidence values in *c*_*C*_, and a zero vector of the same length is inserted into *A*_*S*_, *c*_*SC*_, and *c*_*SF*_, respectively. At last, we compute the covering area of each anchor in *A*_*S*_ and store them in *δ*.

The pseudocode of the merge process can be described as follows.


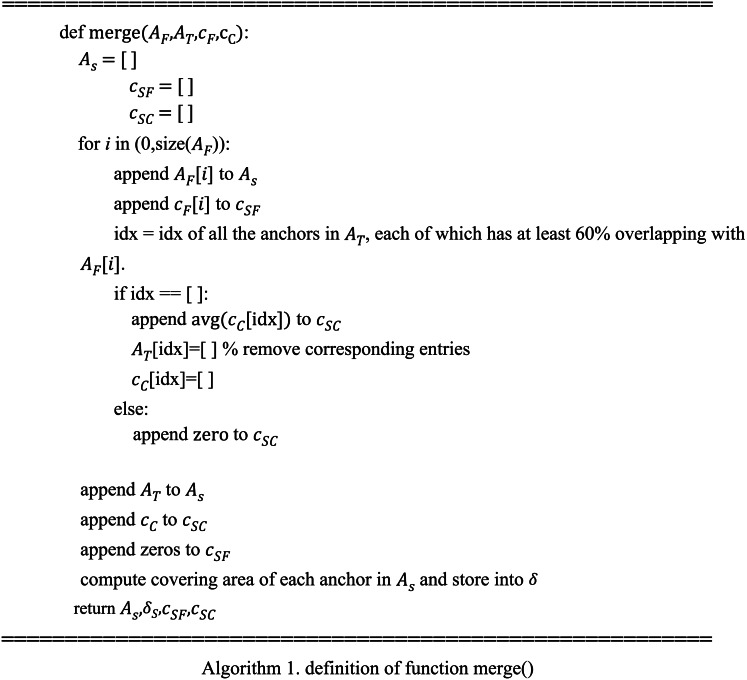


After the data preparation, many ensemble learning methods can be used for model integration. In this work, we consider a basic ensemble model defined as follows

(7) \begin{eqnarray*}S \left( {c}_{SF},{c}_{SC},\delta ,\alpha \right) =\sum _{i}{\alpha }_{i}{h}_{i}({c}_{SF},{c}_{SC},\delta )\end{eqnarray*}

where *α* is the model parameter, *δ* is a vector containing the area of corresponding anchors, and *h*_*i*_() is a classifier. We choose decision trees with maximum depth of two in the experiment, and *i* is ranged from 0 to 1000. The variable *δ* is used here because the two base algorithms are good at identifying relatively large and small objects respectively, and adding covering areas of anchors can help improve the accuracy.

Thus, in the ensemble method, *A*_*S*_ is the anchor lists, and *c*_*S*_ = *S*(*c*_*SF*_, *c*_*SC*_, *δ*, *α*) contains the corresponding confidence values about helmet-wearing. To train this model, we merge the anchor set *A*_*S*_ and the ground-truth set *A* in a similar manner as merging *A*_*F*_ and *A*_*T*_, and we use ${\hat {c}}_{SF}$, ${\hat {c}}_{SC}$ and $\hat {c}$ to represent the corresponding variables after merging. Zeros are filled if the corresponding anchor does not exist before merging. Then, the loss between the identified anchors in *A*_*S*_ and the ground-truth anchors *A* is

(8) \begin{eqnarray*}E(\delta ,\alpha ,{\hat {c}}_{SF},{\hat {c}}_{SC},\hat {c})=\sum _{i=0}^{n}{ \left( S({\hat {c}}_{SF}[i],{\hat {c}}_{SC}[i],\delta ,\alpha )-\hat {c}[i] \right) }^{2}\end{eqnarray*}

where *n* is the total anchors after merging. The optimal value of *α* can be computed by minimizing the error

(9) \begin{eqnarray*}{\alpha }^{\ast }={\text{argmin}}_{\alpha }E(\alpha ,{\hat {c}}_{SF},{\hat {c}}_{SC},\hat {c})\end{eqnarray*}

The whole process can be described by the pseudocode below.


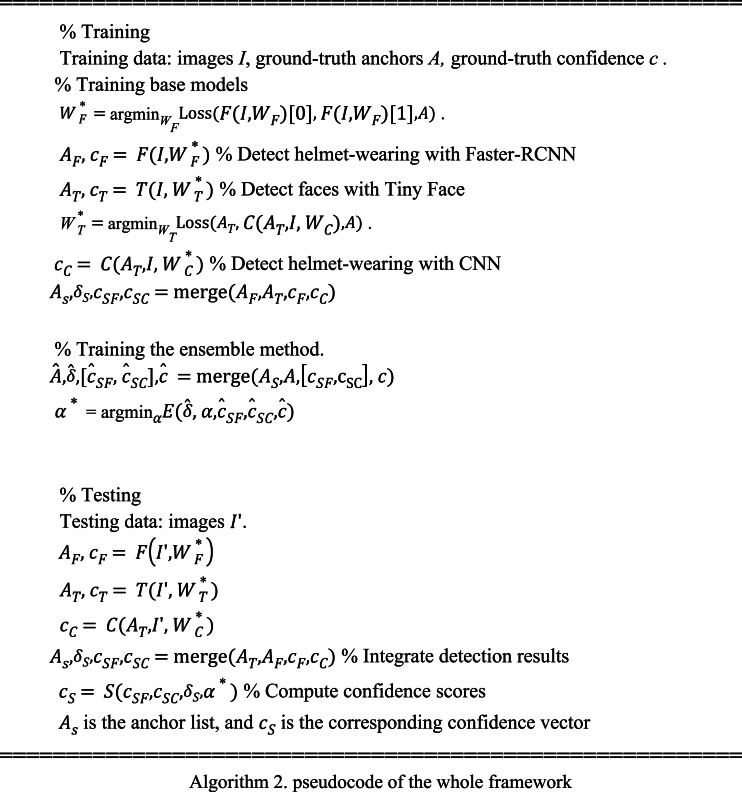


### Experiments

In order to evaluate the performance of our framework, we use five criteria:

(10) \begin{eqnarray*}TPR=m/n\end{eqnarray*}

(11) \begin{eqnarray*}FPR=l/k\end{eqnarray*}

(12) \begin{eqnarray*}RE=m/N\end{eqnarray*}

(13) \begin{eqnarray*}FNR=1-RE\end{eqnarray*}

(14) \begin{eqnarray*}PRE=m/ \left( m+l \right) \end{eqnarray*}

where *TPR* is the true positive rate, *FPR* is the false positive rate, *FNR* is the false negative rate, *RE* is the recall rate, *PRE* is the precision rate, *m* is the correct prediction by models under the current threshold, *n* is the number of parts of the model detection result that are identical to the truth ground, *l* is the false prediction by models under the current threshold, *k* is the number of parts of the model detection result that are different from the truth ground, and *N* is the number of targets that actually exist.

To evaluate our approach, we take the publicly available benchmark data set (Safety Helmet Wearing-Dataset,), containing images from construction sites, roads, workshops, and classrooms. The data set consists of a total of 7,581 images. We use five-fold cross validations for experiments. We randomly divide all the images into five parts. Training set, validation set, and testing set contains 3/5, 1/5, and 1/5 of the total images respectively.

#### Preliminary analysis

**Figure 1:**
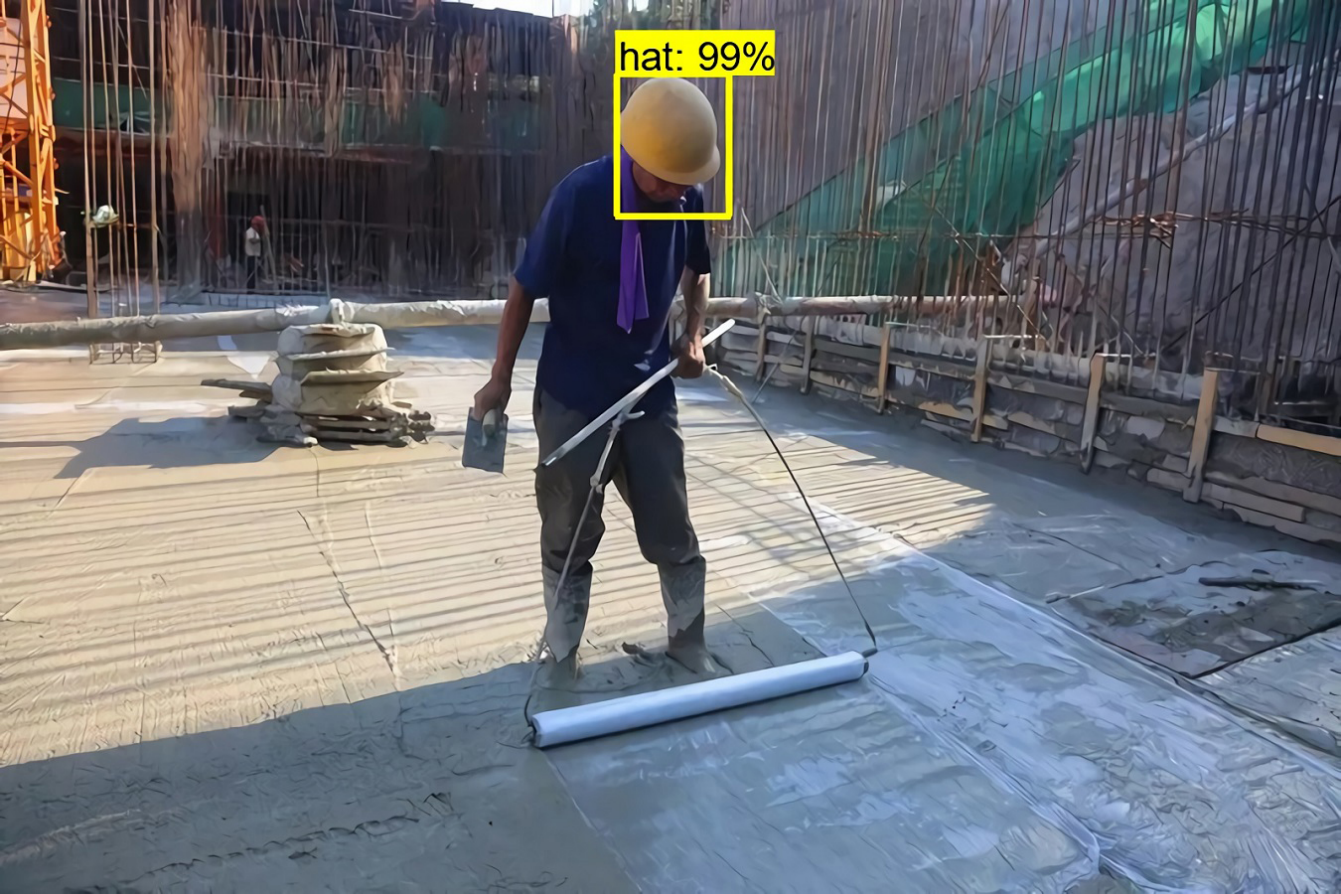
Faster RCNN detecting big faces.

**Figure 2:**
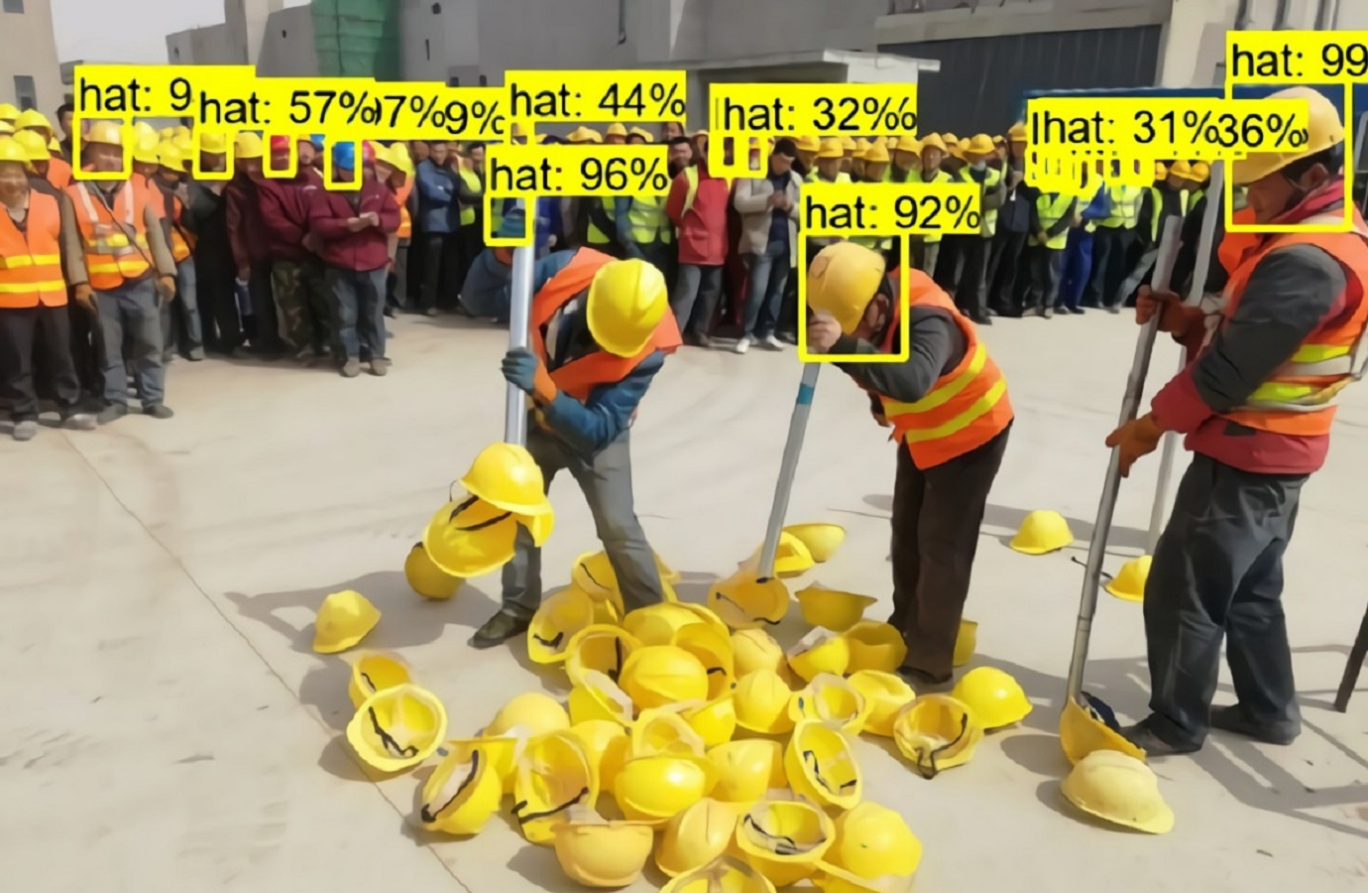
Faster RCNN detecting small faces.

The detection results of Faster RCNN for faces are shown in Figs. 1 and 2. From these two figures, we can see that Faster-RCNN is suitable for detecting large objects, but not finding small ones.

**Figure 3:**
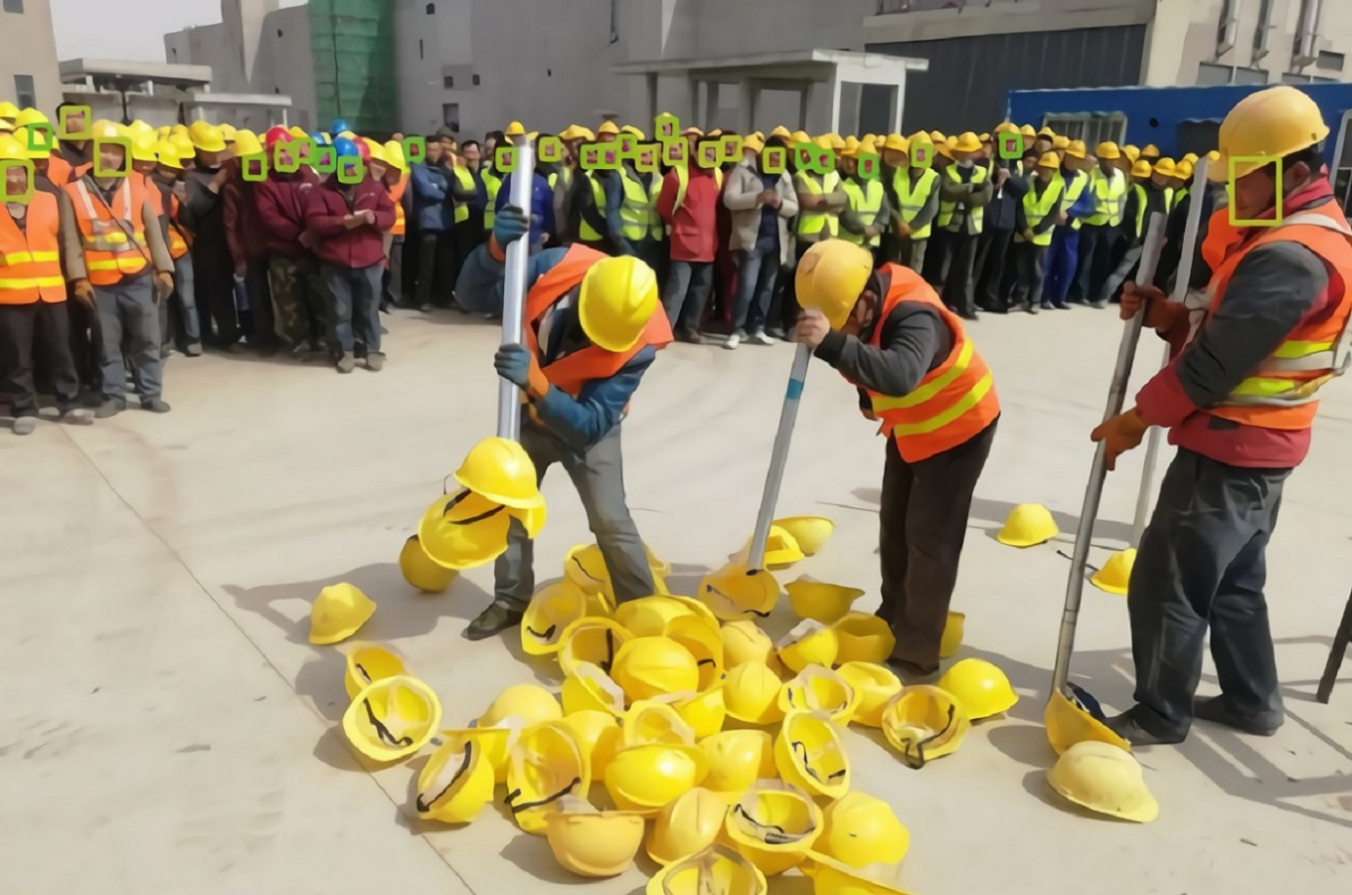
Tiny Face detecting small faces.

The detection results of Tiny Face are shown in Fig. 3. From this result, we can see that Tiny Face is good at finding small faces.

**Figure 4:**
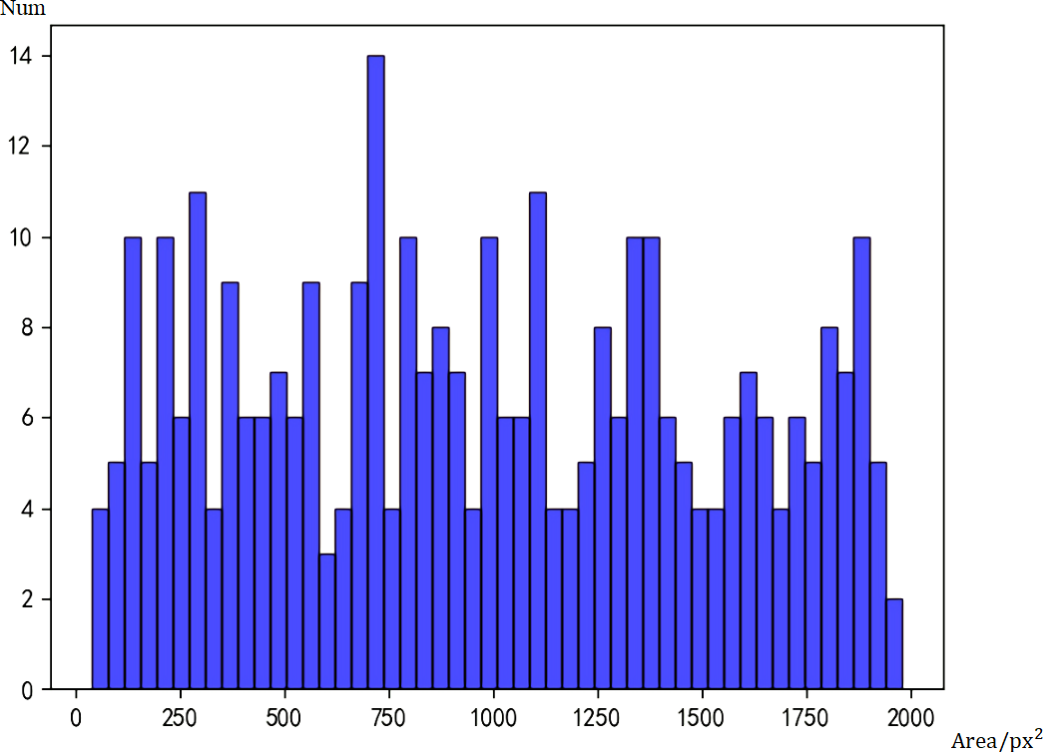
Histogram of real data.

**Figure 5:**
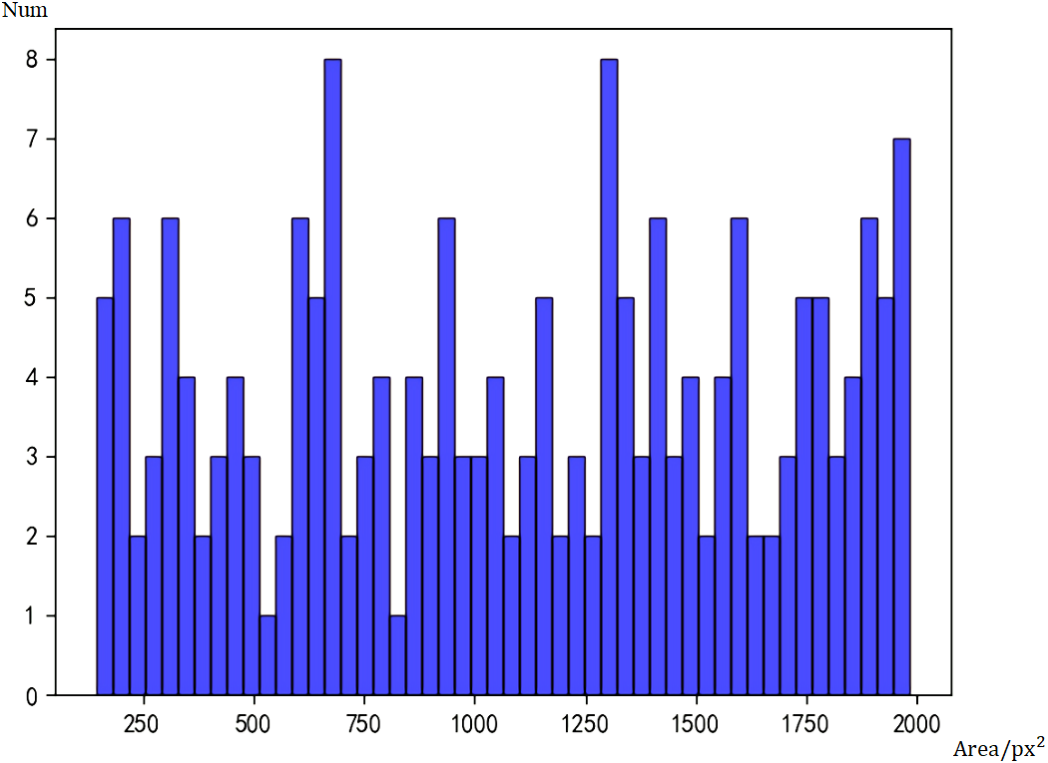
Histogram of face sizes detected by Faster RCNN.

To compare the differences between the two models, we used Faster RCNN and Tiny Face to test the 1000 images from the data set, and count the number of faces of different sizes detected by the two models. Figure 4 is the histogram of real data, and Fig. 5 is the histogram of face sizes detected by Faster RCNN.

Taking the number of pixels (px^2^) as the area measurement, a face with an area smaller than 500px^2^ is defined as a small face, and a face larger than 500px^2^ is defined as a large face. Because of the large area span, the smallest face is only 90px^2^, while the largest face can reach 2000000px^2^. In order to prevent the histograms from crowding together, only faces with an area less than 2000px are shown in the figure.

According to statistics, there are actually 1,568 big faces and 83 small faces. The initial model of Faster RCNN can detect 1,468 big faces and 37 small faces. Under the assumption that the labels are correct, the false negative rate of big faces is 5.2%, and that of small faces is 55.5%. Obviously, the Faster RCNN model has lower accuracy for small faces.

**Figure 6:**
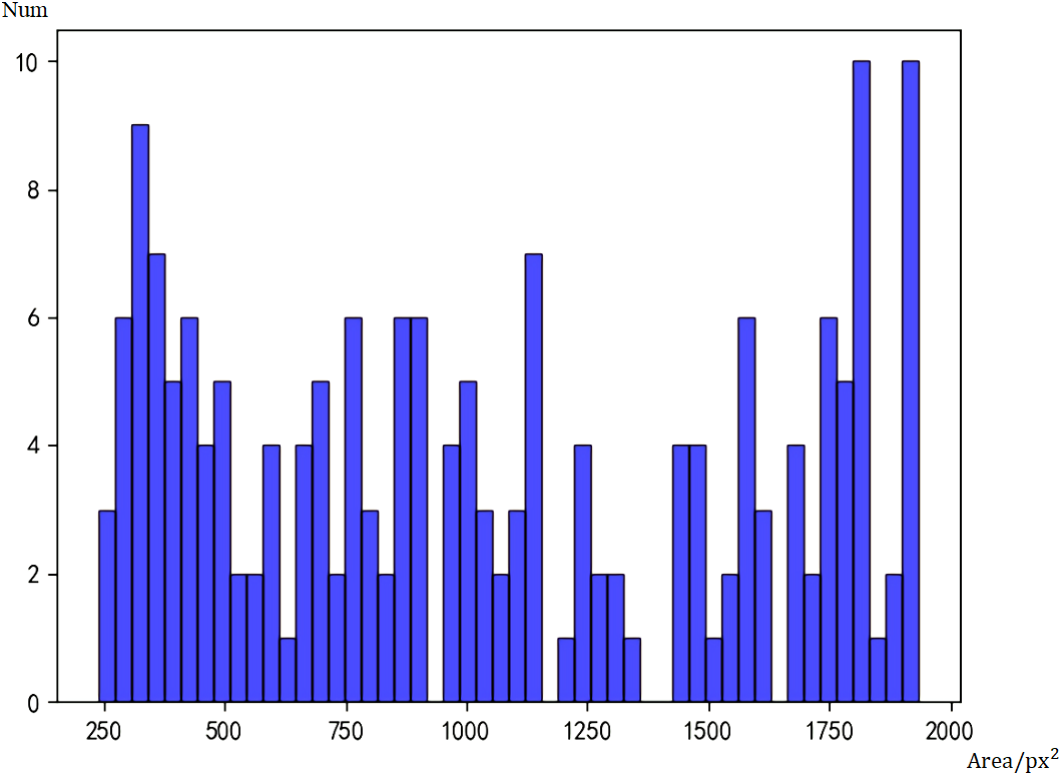
Histogram of face sizes detected by Tiny Face.

Then we performed statistics on Tiny Face and got the histogram of Tiny Face detection results in Fig. 6. Tiny Face can detect 1306 large faces and 44 small faces. The false negative rate for large faces is 16.8%, which is 11.6% higher than Faster RCNN, and the false negative rate for small faces is 47.0%, which is 8.5% lower than Faster RCNN. Although it is only a preliminary model, the model has not been adjusted and the amount of training has been adjusted to improve the accuracy of the model, but it is not difficult to see from the current data that the detection capabilities of the Faster RCNN and Tiny Face models have their own focus. When Faster RCNN detects large faces, it has a great advantage, and Tiny Face’s ability to detect small faces is better than Faster RCNN.

We can find that Faster RCNN has a higher true positive rate for detecting large faces and Tiny Face has a higher true positive rate for detecting small faces. The overall effect can be better if we can combine the two methods.

#### Accuracy of base algorithms for helmet detection

##### Accuracy of $F(I,{W}_{F}^{\ast })$.

In this part, we evaluate the accuracy of $T(I,{W}_{T}^{\ast })$ alone in Algo. 2. Theoretically, the more training steps the model has, the better, but in order to prevent overfitting, we still need to observe the accuracy of the model under different training steps.

In the beginning, we select some images from training dataset to evaluate the model. We trained 5,000 steps and used the model to test the images of the training set, but it was obvious that the effect was not very satisfactory. Because $T(I,{W}_{T}^{\ast })$ is based on Faster RCNN, which has high accuracy, it is easy to miss the mark of small faces. Therefore, the quality of the model can be preliminarily judged by the number of detected targets, and then we gradually increased the number of training steps.

When the number of training steps reaches 20,000 steps, the number of detected targets in the detection results of 1,000 test set images is basically maintained at about 1,300. As the number of training steps increases, the number of detected targets increases slightly. When the number reaches 60,000 steps, the number of detected targets is 1,523. At this time, precision rate of the model is 87.3%,and the recall rate is 85.9%. When the number of training steps reaches 70,000 steps, the number of detected targets is close to 1,700. At this time, the precision rate of the model is 81.2%, and the recall rate is 86.3%. We find that although the recall rate has a slight increase, but the precision rate is much lower, so we chose the model with 60,000 training steps as the final model. See Table 1 for the accuracy of $F(I,{W}_{F}^{\ast })$ under different training steps.

**Table 1:** Relationship between training steps and accuracy.

Steps	Precision rate	Recall rate
5,000	80.0%	72.4%
20,000	84.0%	82.0%
40,000	86.1%	85.1%
60,000	87.3%	85.9%
70,000	81.2%	86.3%

**Figure 7:**
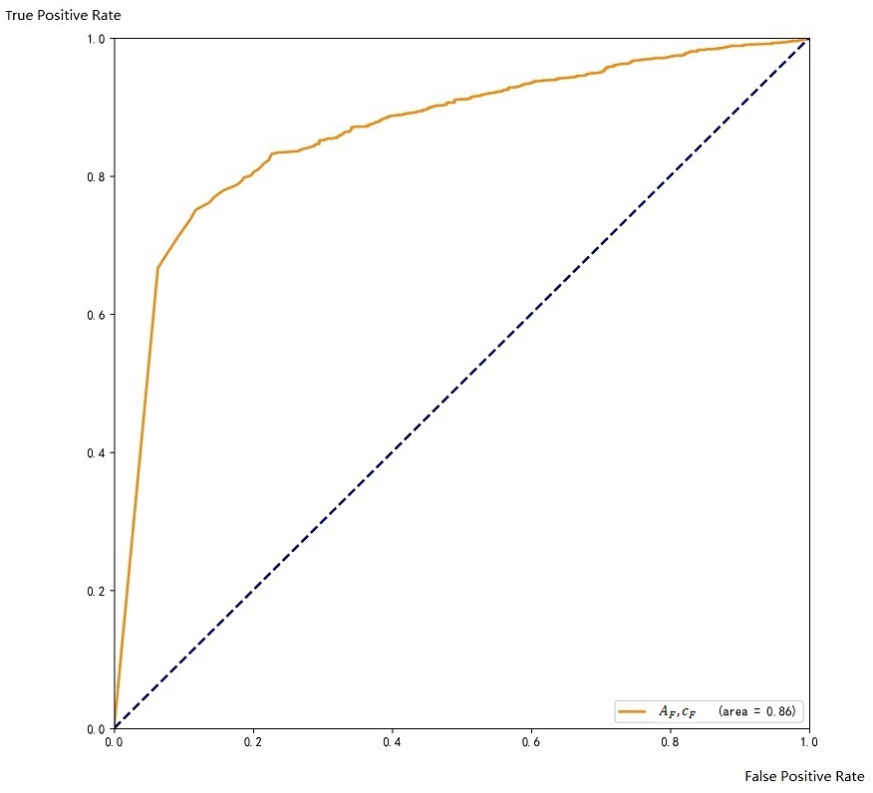
ROC with respect to A

Regarding to the scoring threshold, it is 0.5 by default, which means that when the score is lower than 0.5, the result will be discarded. We successively set the threshold to 0.3, 0.4, 0.5, 0.6, 0.7, and tested the validation data to choose the one that works best. Finally, we found that when the threshold is 0.6, the precision rate of the test result is 87.3%, and the recall rate is 85.9%, which is better than other thresholds. After comprehensive consideration, we finally keep 0.6 as the threshold for the ensemble. The ROC curve on the training set is shown in Fig. 7.

When training this model, in order to distinguish whether an individual wearing a helmet, we use two labels: people wearing and without wearing a helmet. It makes the final trained model more accurately distinguish whether the target wears a helmet.

##### Accuracy of $T(I,{W}_{T}^{\ast })$.

In this part, we consider the accuracy of $T(I,{W}_{T}^{\ast })$ alone in Algo. 2. It is basically a trained Tiny Face model. We lowered the scoring threshold of Tiny Face to 0.5, requiring the Tiny Face model to be able to determine the location of the small face, and it does not need it to accurately return the scoring value. The precision rate of face detection was 85.6%, and the recall rate was 69.4%.

##### Accuracy of $C({A}_{T},I,{W}_{C}^{\ast })$.

In this part, we consider the accuracy of $C({A}_{T},I,{W}_{C}^{\ast })$ alone in Algo. 2. Function $C({A}_{T},I,{W}_{C}^{\ast })$ is basically a CNN model, which requires only one target in an image, so we select over 2,000 images from the training set, cropped the target according to the corresponding anchor labels and get 20,000 images with only one target in each image. We select 18,000 images as training data for CNN, and the other 2,000 images as a validation set to detect the accuracy of CNN, also the cropped images are divided into two sets, people wearing helmets and without wearing helmets. In addition, we rotate some images to get richer training samples.

With cross-validation, we choose to use four pairs of convolution and pooling layers, of which the first layer and the size of the convolution kernel of the second convolution layer are [5,5], and the size of the convolution kernel of the third and fourth convolution layers is [3,3]. The precision rate of the final two-class CNN reached 90.3% when we use it to test the validation set of CNN.

**Figure 8:**
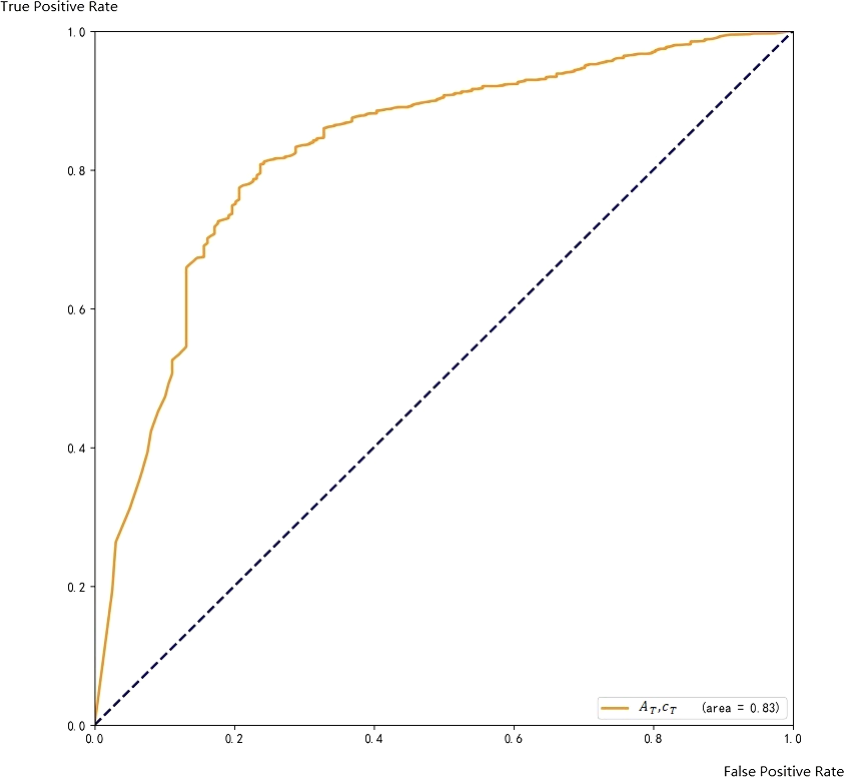
ROC with respect to A_T and c_C.

The ROC curve on the training set is shown in Fig. 8.

##### Accuracy of Ensemble Method $S \left( {c}_{SF},{c}_{SC},{\alpha }^{\ast } \right) $.

The ROC curve coverage areas of Faster CNN and *A*_*T*_, *c*_*C*_ are 0.86and 0.83, respectively. The ensemble method can further improve the accuracy of the final result.

**Figure 9:**
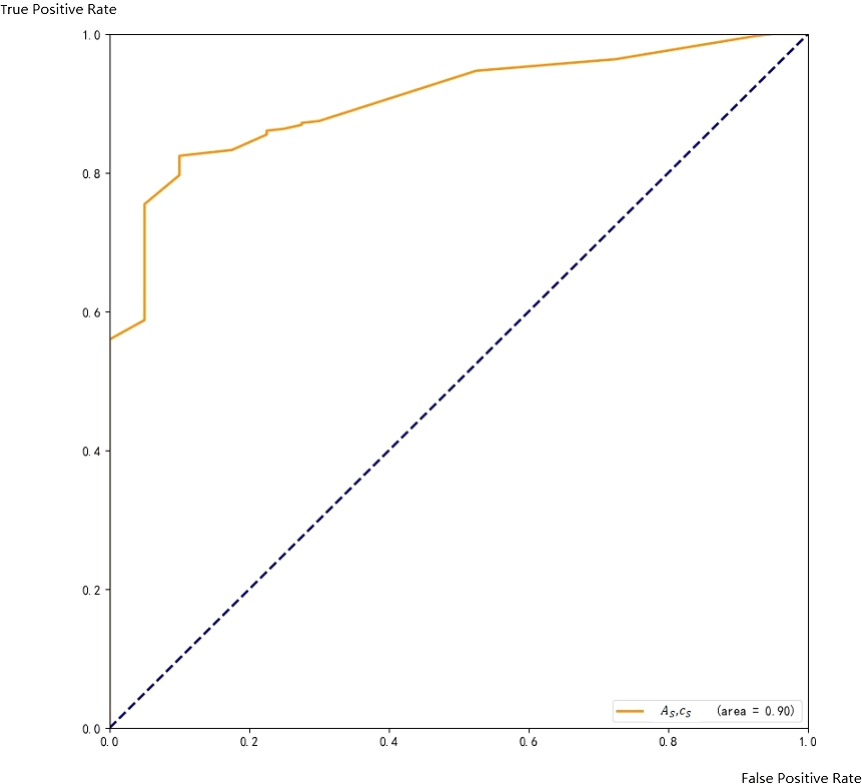
ROC with respect to A_S and c_S <!–[if !msEquation]–><!–[endif]–>.

Among the data, *c*_*F*_ and *c*_*C*_ are the results from two base methods, respectively, and the area is the size of the target frame. Obviously, *c*_*F*_ and *c*_*C*_ can be used as the characteristic values of the ensemble method. We test the trained model, and the area under the ROC curve coverage is larger, becoming 0.90. The ROC curve on the training set is shown in Fig. 9.

Obviously, the ROC curve covered by the ensemble method has the largest coverage area, which proves that the ensemble method is effective in our model.

**Figure 10:**
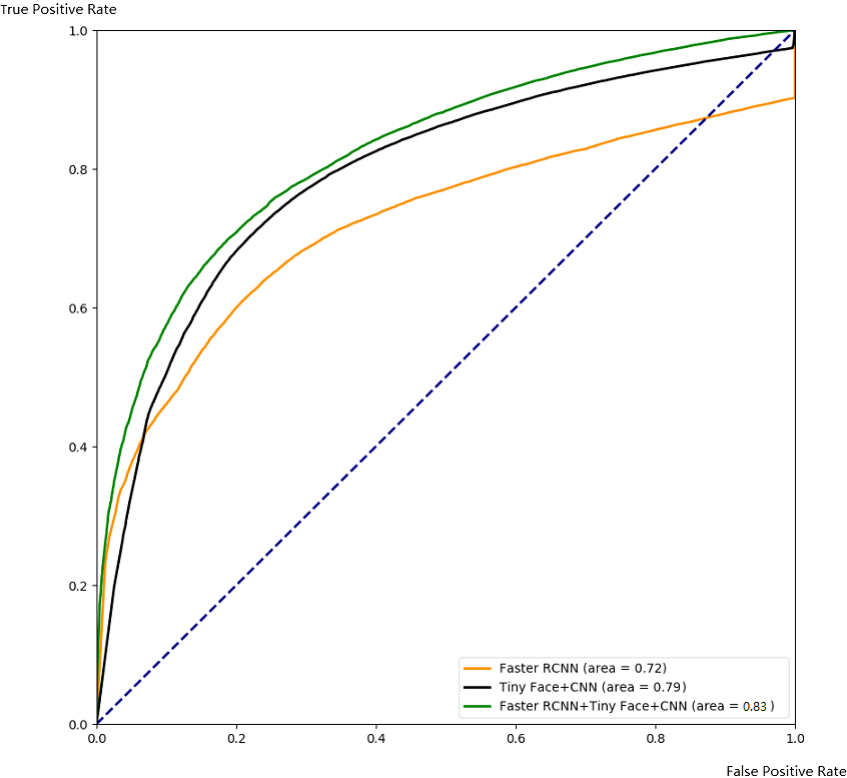
Comparison with ROC.

**Figure 11:**
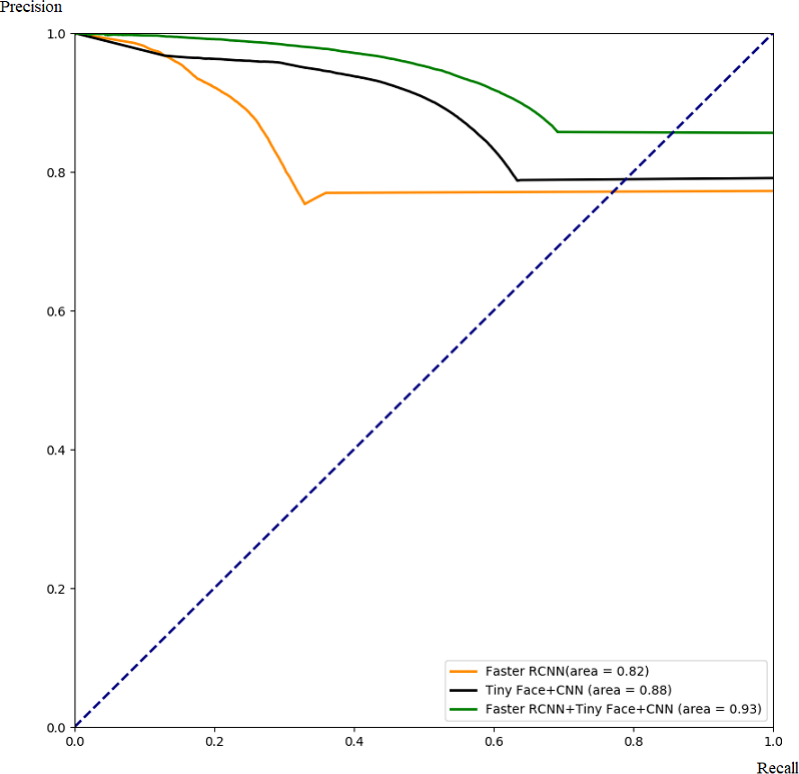
Comparison with PR.

##### Comparison of different algorithms.

In this part, we demonstrate the effectiveness of our ensemble framework by combining Faster RCNN and Tiny Face+CNN together with ROC curve and PR curve. The ROC and PR curves are calculated from testing results through 5-fold cross-validation as Figs. 10 and 11. From Figs. 10 and 11, we can see that combination with our framework (in green) is better than single algorithms (in black and orange). Our framework can also gain the largest area under the ROC curve (0.83) in Fig. 10, and the largest area under the PR curve (0.93), namely the mAP score. It means our framework works best on average over all the possible threshold choices.

Tables 2 and 3 reveals a similar phenomenon when a reasonable threshold is chosen. It indicates that, with a well-chosen threshold, our framework works better than others in terms of TPR, FPR,FNR, precision, and recall.

**Table 2:** Comparison with TPR, FPR, FNR.

Algorithm	True positive rate	False positive rate	False negative rate
Faster RCNN	74.7%	43.1%	72.7%
TinyFace + CNN	73.8%	25.8%	51.9%
Faster RCNN + Tiny Face + CNN	75.6%	18.3%	42.5%

**Table 3:** Comparison with precision and recall.

Algorithm	Precision	Recall
Faster RCNN	85.4%	27.3%
Tiny Face + CNN	91.5%	48.1%
Faster RCNN + Tiny Face + CNN	92.5%	57.5%

**Figure 12:**
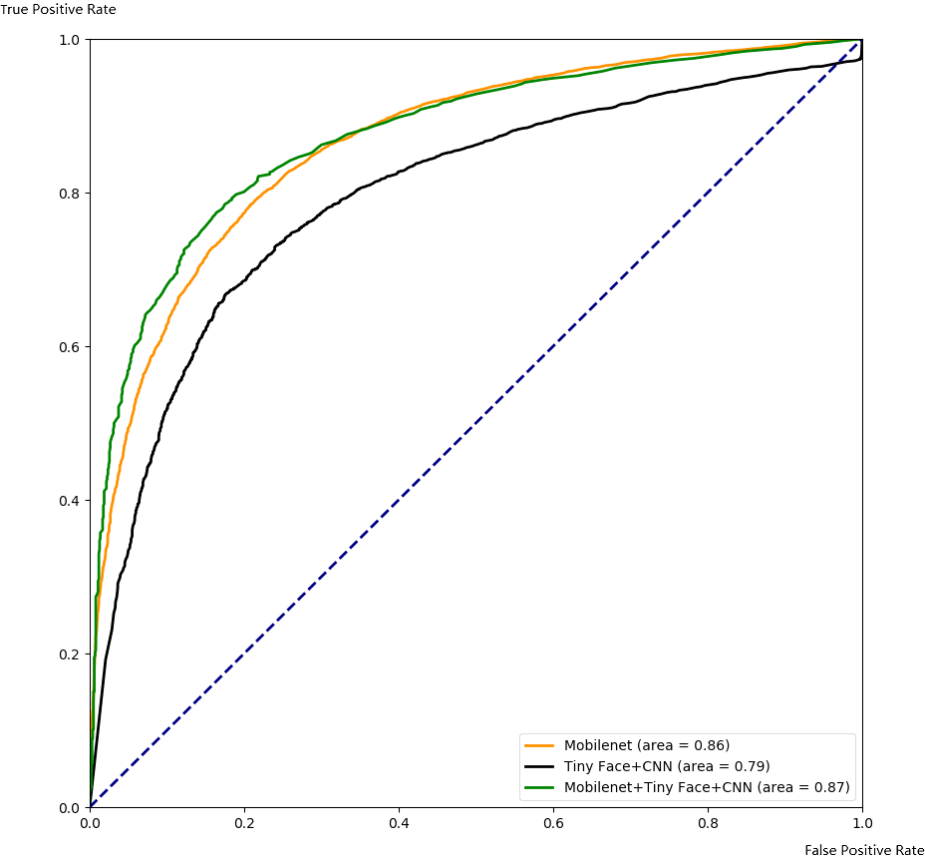
Comparison with ROC for integrating Mobilenet and Tiny Face.

**Figure 13:**
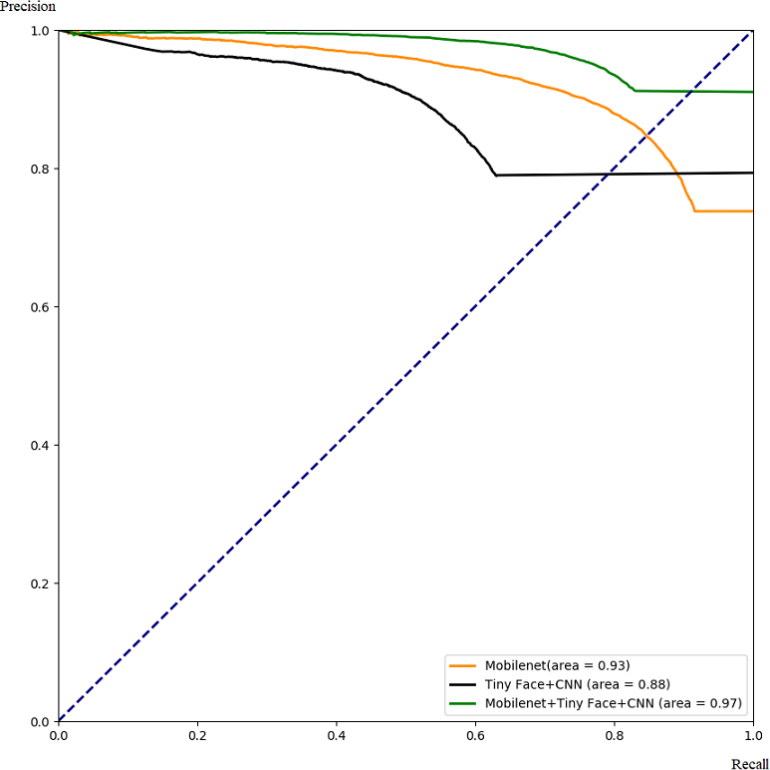
Comparison with PR for integrating Mobilenet and Tiny Face.

Our framework can also be used to integrate other complementary deep learning methods to improve their performance. As an example, we use our framework to combine Mobilenet and TinyFace+CNN, and compare the integrated results with single algorithms. The performance is shown in Figs. 12 and 13. Similar to the previous case, the algorithm performance is generally improved. Our framework also works well when a specific threshold is chosen, as shown in Tables 4 and 5.

**Table 4:** Comparison with TPR, FPR, FNR for integrating Mobilenet and Tiny Face.

Algorithm	True positive rate	False positive rate	False negative rate
Mobilenet	74.3%	17.4%	32.0%
TinyFace + CNN	73.3%	25.2%	52.5%
Mobilenet + Tiny Face + CNN	80.0%	17.2%	35.2%

**Table 5:** Comparison with Precision and Recall for integrating Mobilenet and Tiny Face.

Algorithm	Precision	Recall
Mobilenet	92.0%	69.4%
Tiny Face + CNN	91.9%	47.7%
Mobilenet + Tiny Face + CNN	94.7%	77.7%

Through these experiments, we can find that the integrated framework for two complementary models can improve the performance of single algorithms by increasing the true positive rate, the precision rate, and the recall rate, while reducing the false positive rate and false negative rate.

## Discussion

The detection accuracy of a single model is usually not a satisfactory, so we use an ensemble method to integrate models to get better results. Considering complementary behaviors of different algorithms, using an ensemble method for integration can effectively improve the accuracy of the detection results. For example, through our experiments, we can use Tiny Face model with CNN to overcome the shortcomings that the Faster RCNN model possesses when detecting small faces. Although the proportion of small faces in the test set of this experiment is not very large, the missing rate is still one percent lower than that of a single model. In the test set with a large proportion of small faces, the detection accuracy of the integrated model can be improved further.

## Conclusion

When the detection accuracy of a single deep learning model could not meet the demand for helmet-wearing detection, we can integrate a complementary model with it to get better results. In addition, our framework can make single algorithms more robust to data sets from different scenarios, because it can utilize the advantage of the complementary algorithms.

By analyzing a variety of object detection models, we find that many models are difficult to achieve high-precision for helmet-wearing detection in different scenarios. Therefore, we carefully select two complementary base models and add additional modules to make them suitable for helmet-wearing detection. We ensemble the base models and build a more powerful helmet-wearing detection algorithm to further improve the detection capability. Our approach can be accelerated by GPU and be deployed on distributed computers to reduce processing time, and thus, can be useful in real-world scenarios. In the future, the model can also be extended by integrating additional features or models and upgraded to mixed neural network models.

##  Supplemental Information

10.7717/peerj-cs.311/supp-1 Supplemental Information 1 Code implementations for the paper Base models and the ensemble models.

## Additional Information and Declarations

### Data Availability

The following information was supplied regarding data availability: Code is available as a Supplemental File. The data set is available at GitHub: https://github.com/njvisionpower/Safety-Helmet-Wearing-Dataset.
